# Giant inflammatory fibroid polyp of ileum causing intussusception: a case report

**DOI:** 10.4076/1757-1626-2-8616

**Published:** 2009-08-12

**Authors:** Sami Akbulut, Mert Mahsuni Sevinc, Bahri Cakabay, Sule Bakir, Ayhan Senol

**Affiliations:** 1 Department of Surgery, Diyarbakir Education and Research HospitalDiyarbakir 21400Turkey; 2 Department of Pathology, Diyarbakir Education and Research HospitalDiyarbakir 21400Turkey; 3 Department of Radiology, Diyarbakir Education and Research HospitalDiyarbakir 21400Turkey

## Abstract

Inflammatory fibroid polyps are rare, localized, non-neoplastic lesions originating in the submucosa of the gastrointestinal tract. Intussusception due to inflammatory fibroid polyps is uncommon; moreover, ileo-ileal intussusception has only rarely been reported. Here, we report an 11 × 7 cm giant inflammatory fibroid polyp of the small bowel that presented as intussusception in a 73-year-old woman. Ultrasonography demonstrated a solid, homogeneous, echogenic mass surrounded by the typical mural layers of an invaginated ileum. The immunohistopathological diagnosis after segmental ileal resection was an ileal inflammatory fibroid polyp. Although encountered rarely in adults, physicians should be aware of invagination and consider it in each case of acute abdomen because of the wide spectrum of clinical settings.

## Introduction

Intussusception occurs when a more proximal portion of bowel invaginates into more distal bowel [1]. Adult intussusception is relatively rare, constituting only 1% of patients with bowel obstructions [2]. The cause of intussusception in children differs from that in adults. Adult invagination is mostly caused by tumors [3] and 80% of the tumors associated with small bowel intussusception are benign [3,4]. Lipoma is the most common benign tumor in intussusception. Inflammatory fibroid polyps (IFP) rarely cause ileal intussusception. Here, we report an unusual case of ileo-ileal intussusception caused by an IFP. In addition, this is the third largest IFP reported in the English literature [5,6].

## Case presentation

**Figure 1.**
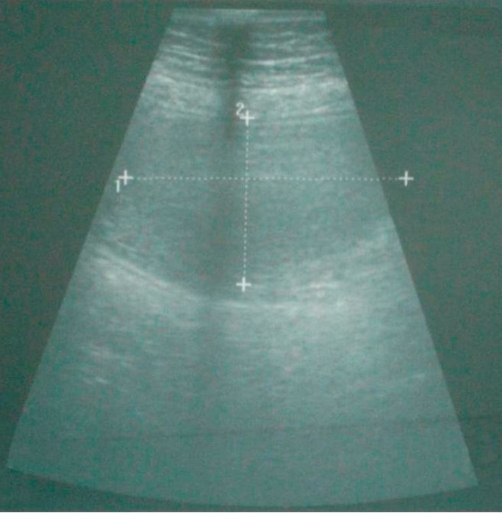
Ultrasound examination: a transverse section of an ileal loop shows an echogenic 7-cm intraluminal mass.

**Figure 2.**
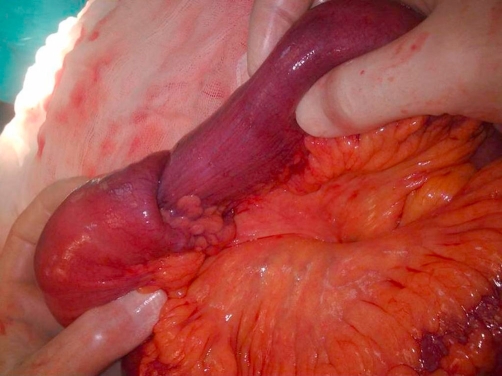
The view of the invaginated segments.

**Figure 3.**
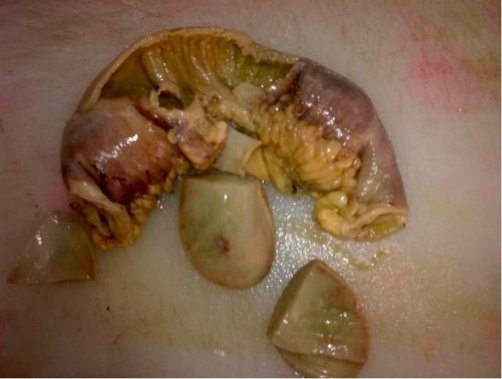
The resected ileal segments with the polyp.

A 73-year-old Turkish woman was admitted to our emergency unit suffering from abdominal pain, nausea and vomiting, constipation, no passage of gas or feces, and abdominal distension for 5 days. Laboratory investigations showed the following: blood urea nitrogen, 27 mg/dl; creatinine, 0.9 mg/dl; and C-reactive protein, 72 mg/L. The blood cell count revealed leukocytosis at 18,000/μl, hemoglobin of 13.7 g/dl, and a platelet count of 524,000/μl. Other serum parameters were within normal limits. The physical examination revealed muscular guarding and rebound tenderness in the periumbilical region. An upright plain abdominal film revealed small bowel obstruction with marked small bowel air-fluid levels. Abdominal ultrasound revealed a target lesion in a small bowel segment, indicating intussusception (Figure 1). The patient underwent an emergency laparotomy via a midline incision. On exploration, an ileo-ileal intussusception was found 100 cm proximal to the ileocecal valve. The intussuscepted intestinal segments were obstructing the lumen, causing dilatation in the intestine proximal to the intussusception (Figure 2 and Figure 3). We performed a segmental small bowel resection and an end-to-end anastomosis. The patient had an uneventful postoperative course and was discharged on the sixth postoperative day. The pathology examination showed an 11 × 7 cm inflammatory fibroid polyp composed of an edematous stroma containing spindle-shaped stromal cells, lymphoid nodules, and eosinophils.

## Discussion

Intussusception develops due to a difference in motility between two intestinal parts. One segment (the intussusceptum) enters a neighboring one (the intussusceptiens) [7]. Barbette of Amsterdam first reported intussusception in 1674 [8]. Malignant or benign lesions, usually appearing at the head of the invagination, cause 65% of all adult intussusceptions. Malignant lesions are found in 14 ~ 47% of cases occurring in the small intestine. Benign tumors as the lead points of an intussusception include lipomas, leiomyomas, neurofibromas, adenomas, and inflammatory fibroid polyps [9].

Inflammatory fibroid polyps are rare, benign, tumor-like lesions of the gastrointestinal tract. The lesion was first described by Vanek in 1949. Most frequently, they are localized in the gastric antrum, but can develop anywhere in the gastrointestinal tract. In the small intestine, the ileum is the most common site where these polyps cause intussusception. An IFP is a benign reactive lesion that occurs predominantly in adults. Most IFPs are polypoid masses smaller than 5 cm, although sizes up to 20 cm have been reported [10-12]. In the literature, the average age of intussusception in adults is 38.6 ~ 57.5 years. The gender distribution is roughly equal [13]. To our knowledge, our patient is the second oldest to develop invagination due to an IFP [14].

A preoperative diagnosis of intussusception is rare, but can be made on finding a palpable mass in the abdomen or with the use of imaging techniques [15]. The primary imaging modality of choice is ultrasound scanning, which enables the diagnosis or exclusion of intussusception with a sensitivity of 98 ~ 100%, specificity of 88%, and negative predictive value of 100% [16]. The majority of intussusceptions are diagnosed at surgery. In our case, the preoperative clinical findings and ultrasonic images suggested invagination. The ultrasound images of the small bowel lumen showed a solid mass 7 cm in diameter. The clinical presentation in adult intussusception is often chronic, and most patients present with non-specific symptoms that are suggestive of intestinal obstruction. Abdominal pain is the most common symptom, followed by vomiting and nausea [7]. The optimal management of adult intussusception remains controversial. Exploratory laparotomy is frequently recommended as a treatment for fibroid polyps. The lesion seems to lack malignant potential and recurrence of the polyp has been reported only once [7]. Our patient has had no recurrence after follow-up for 18 months.

Morphologically, IFPs can mimic several other tumor and non-tumor processes of the gastrointestinal tract, including inflammatory myofibroblastic tumors, eosinophilic gastroenteritis, gastrointestinal stromal tumors (GISTs), and other mesenchymal lesions. Sometimes differentiation is difficult, especially differentiation between inflammatory fibroid polyps and GIST [12]. In our case, the immunohistochemical examination showed spindle-shaped cells in the circumference of small blood vessels expressing vimentin, but not CD34, CD117, or SMA.

In conclusion, the recommended treatment of adult intestinal invagination is surgical resection of the intestinal segments involved. This case report demonstrates that intussusception, although rare in adults, should be considered in the differential diagnosis of abdominal pain.

## Abbreviations

GIST: gastrointestinal stromal tumor
IFP: inflammatory fibroid polyps
